# Body composition, water turnover and physical activity among women in Narok County, Kenya

**DOI:** 10.1186/1471-2458-14-1212

**Published:** 2014-11-24

**Authors:** Susan Keino, Bart van den Borne, Guy Plasqui

**Affiliations:** Department of Health Education & Promotion, Faculty of Medicine & Life Sciences, Maastricht University, P.O BOX 616, 6200 MD Maastricht, The Netherlands; Department of Epidemiology & Nutrition, School for Public Health, Moi University, P.O Box 4606, 30100 Eldoret, Kenya; Department of Human Biology, School for Nutrition, Toxicology & Metabolism, Maastricht University Medical Centre, P.O BOX 616, 6200 MD Maastricht, The Netherlands

**Keywords:** Nutritional status, Sub-Saharan Africa, Water turnover, Deuterium dilution, Accelerometry

## Abstract

**Background:**

In developing countries where access to water and food is not guaranteed, women may have to travel long distances or engage in intense physical activities to gather food. This may compromise their water requirements and overall nutritional status. The aim of the study was to determine water turnover, physical activity and body composition among women in Kenya and to describe the differences between rural and urban Kenyan women.

**Methods:**

Thirty women from Narok County who were not pregnant at the time of the study were recruited. Body mass index (BMI) was calculated by dividing weight in kilograms by height in meters squared. Deuterium dilution was used to determine total body water (TBW) and water turnover was measured from deuterium elimination. Fat-free mass (FFM) was calculated by assuming a constant hydration fraction of 73.2%. Accelerometers (Actigraph GT3X) were used to assess physical activity and expressed as Vector magnitude counts per day (VM/day). Simple and multiple linear regressions were used to define the determinants of water turnover.

**Results:**

Mean BMI was 23.4 ± 4.1 and 21.5 ± 3.8 among rural and urban women respectively. The prevalence of overweight (BMI > 25 kg/m^2^) was 24.1% and of underweight (BMI < 18.4 kg/m^2^) was 25%. The mean total body water (TBW) was 29.3 ± 4.2 liters (L) and water turnover was 3.2 ± 0.8 liters per day (L/day). Water loss was positively associated with BMI (R^2^ = .45, *p* < 0.001, *n* = 28) and Fat mass index (FMI) (R^2^ = .41, *p* < 0.001, *n* = 28). Water loss was also positively associated with physical activity (PA) (R^2^ = .25, *p* < 0.05, *n* = 22). Multiple regression analysis showed that physical activity in addition to BMI in the model explained an additional 15% of the variation in water turnover (r^2^ = 0.53, *p* < 0.05; ∆r^2^ = 0.15, *p* < 0.05, *n* = 22) compared to BMI alone (r^2^ = 0.38, *p* < 0.005 *n* = 22).

**Conclusion:**

BMI together with physical activity were the strongest predictors of water loss.

## Background

Physical activity in most sub-Saharan Africa is part and parcel of the daily routine of walking in searching for water and gathering food [1, 2] and physical activity is more common in the rural compared to the urban areas since the rural populations rely on walking as a means of transport and often have labor-intense agricultural activities as their main occupation [2]. During increased physical activity, there is increased sweating and breathing which contributes to body water loss [3]. Water is an essential nutrient in all known forms of life [4] and it is the principal chemical constituent of the human body [5]. Variability in total body water in free living humans is primarily due to differences in body composition [5]. However the impact of physical activity on water loss among individuals living in the warm climatic conditions of Africa is not well understood and there is limited data of studies done in Africa. A study in the Netherlands found mean water loss among women to be 3.1 L/d during summer and 3.0 L/d in winter and the reason for the high water loss during summer was a result of sweating and increased physical activity during this season [6]. In that study, no relation was found between water loss and physical activity among women. Another study showed that the average water loss at high altitude was found to be 3.3 ± 0.6 L/d [7] and 3.0 ± 0.6 L/d in sedentary subjects at over 6000 m above sea level [8]. The impact of physical activity (PA) on water loss among women in Kenya is crucial since women are the primary caregivers within homes [9] resulting in high workloads and energetic demands [10]. Thus fluid loss can compromise their nutritional status and lead to reduced work performance due to dehydration and general apathy [11].

Body composition could affect physical activity levels (PAL) in that individuals with a higher fat deposition have been found to be less physically active than normal-weight individuals [12]. The relationship between body composition and water turnover has been studied in different populations. A study on women in New Zealand found water turnover to be strongly correlated with body weight and fat free mass (FFM) [13]. Another study found that lean body mass (LBM) and fat mass (FM) influenced water turnover in females and not in males [14]. However, the use of body mass index (BMI) to assess body composition is faced with shortcomings as described by Schutz et *al*. [15] and Lu et *al*. [16]. To make up for this shortcoming, there is need to measure FM and FFM and adjusting it for height^2^ to get fat mass index (FMI) and fat free mass index (FFMI), since these measures may have advantages in assessing adiposity. Studies in Kenya that used deuterium dilution to assess body composition among women are limited and one study on lactating mothers found the average percentage FM to be 26.1 ± 5.4 and FFM as 41.3 ± 5.1 kg [17]. However, data on the relationship between water turnover and FMI and FFMI among black race is not available to our knowledge making it difficult to make any recommendations regarding water requirements for people living in communities in warm climates like Kenya. Therefore the aim of this study was to assess water turnover, and to determine the effect of body composition and physical activity on water loss in Kenyan women. Another aim was to describe the difference in water turnover, body composition and physical activity among rural and urban Kenyan women.

## Methods

### Subjects

A total of 30 women aged 15–45 years old in Narok County, Kenya were sampled from two clusters namely *Rotian* and *Majengo* which are both rural and urban settings respectively to participate in this study. The study group included 10 women from the rural and 20 from the urban Narok North Country in Kenya. The inclusion criteria were that the woman had to have a child less than five years old and were not pregnant at the time of data collection. One subject declined to participate after recruitment and another subject was left out of the analysis because of violation of the protocol resulting in invalid numbers for total body water and water turnover leaving a total of 28 women. After the accelerometer data was downloaded and validated, 22 women wore the accelerometer for at least 2 days and 600 minutes per day was set as a minimum criteria for inclusion of the person for data analysis. Prospective participants received verbal information about the nature and purpose of the study and those who agreed to participate signed or thumb-printed on the consent form. The procedures for this study were approved by the Institute for Research and Ethics Committee (IREC) of the Moi Teaching and Referral Hospital (MTRH) and Moi University.

### Anthropometry

Body mass (BM) was measured to the nearest 0.1 kg with a mechanical scale (SECA 762, Chico, CA, USA). Height was measured to the nearest 0.1 cm with a stadiometer (SECA 214, Chico, CA, USA). Body mass index (BMI) was calculated by dividing weight in kilograms by height in meters squared (BMI = kg/m^2^). The classifications for BMI according to WHO are BMI < 18.5 kg/m^2^ for underweight; BMI = 18.5-24.9 kg/m^2^ for normal and BMI > 25 kg/m^2^ for overweight.

### Body composition and water loss

Total body water (TBW) was measured using Deuterium oxide dilution technique according to a slightly modified Maastricht protocol [18] i.e. water turnover was measured from deuterium elimination over a 3-day period instead of the 7-day period that was described by Goris et al. [19]. In the morning participants drank a dose of 70 ml deuterium oxide water (^2^H_2_O) water (70 ml water is enriched with 5 atom percentage excess, so approximately 3.5 g ^2^H) resulting in an enrichment of 50–100 ppm above background levels (~150 ppm). Given the low price of the deuterium isotope, the same dose of deuterium was used for all women, thereby reducing preparation time. Subsequently, two urine samples were collected 6 hours after dosing and one sample after the third day. Water loss was calculated from deuterium elimination by using the isotopic enrichment above baseline from the initial and final samples of the 3 day observation interval [20] Fat free mass (FFM) was calculated from TBW assuming a 73.2% constant FFM hydration fraction. FFM and FM indices (FFMI and FMI) were calculated as proposed by Schutz et *al*. [15] using concepts equivalent to the BMI calculations as shown in the following definition FFMI = FFM/height (m)^2^ and FMI = FM/height (m)^2^. Thus, BMI = FFMI + FMI.

### Physical activity

Actigraph GTX3 accelerometers (Actigraph, Pensacola, FL, USA) were used to determine physical activity. The tri-axial accelerometer measures 4.6 cm × 3.3 cm × 1.5 cm and weighs 19 g and is a validated tool that has shown to be reliable for the assessment of PA [21]. It was attached to the lower back by means of an elastic belt and worn all the time for a period of 2–4 consecutive days except during sleep and while taking a bath or shower. The GT3X measures accelerations of the trunk (counts) in the antero-posterior, medio-lateral and longitudinal axes, otherwise referred to as X, Y & Z axes. The GT3X is designed to enable data storage for 30 days (with fully charged battery) and for optimal wearing comfort in order not to interfere with daily activities. The data was downloaded using the Actilife software and total physical activity was expressed as Vector magnitude counts per day (VM/day). Time spent on different intensities of physical activities were obtained from the cut-offs of Troiano et *al*., which are included in the Actilife software and based on the activity counts of the vertical axis [22]. Actilife software was used to determine the non-wear time and was based on the vector magnitude where at least 60 minutes of consecutive zero counts were required to determine a period as non-wear time [22]. Only days with at least 600 min of wear time were included.

### Data analyses

Microsoft© Excel 2010 for windows was used to enter the data and SPSS version 19 was used to analyze the data. Descriptive measures were calculated to describe the sample by use of percentages, means and standard deviations. Simple linear regression and multiple regression analyses were used to assess the relationship between water turnover (L/d) as the dependent variable and values obtained from body composition (FFMI, FMI, BMI, BM and TBW) and those obtained from physical activity (VM/d) as the independent variables.

## Results

### Body composition and water turnover

A total of 28 women were included in the study and the mean age was 25 ± 6.0 (Table 1). Mean BM was 58.4 ± 11.0 kg and 56.2 ± 11.4 kg among rural and urban women respectively, while mean BMI was 23.4 ± 4.1 among rural women and 21.5 ± 3.8 among urban women. There was no significant difference in the means for the two groups in terms of height, body mass, and body mass index. Of the total women, 24.1% were overweight and 25% were underweight. The average TBW was 29.3 ± 4.2 L, while average water turnover was 3.2 ± 0.8 L/day (Table 2). The body composition results indicate that there was no significant difference in the TBW, L/day, FM, FFM, FMI and FFMI means of the rural and urban groups.

**Table 1:** **Anthropometric measurements of rural and urban women in Narok County**

	Population mean SD*n*(28)	Rural mean*n*(10)	Urban mean*n*(18)
Age	25 ± 6.1	26.7 ± 7.6	24.1 ± 5.2
Height (m)	1.60 ± 0.1	1.58 ± 0.0	1.61 ± 0.1
BM (kg)	57.0 ± 11.1	58.4 ± 11.0	56.2 ± 11.4
BMI (kg/m^2^)	22.2 ± 3.9	23.4 ± 4.1	21.5 ± 3.8

*Abbreviations:* m-meters, kg- kilograms BM-body mass, BMI – body mass index, SD-standard deviation.

**Table 2:** **Body composition and water turnover of rural and urban women in Narok County, Kenya**

	Population mean (SD)*n*(28)	Rural mean (SD)*n*(10)	Urban mean (SD)*n*(18)
TBW (L)	29.3 ± 4.2	29.4 ± 3.5	29.2 ± 4.6
Water turnover (L/day)	3.2 ± 0.8	3.3 ± 1.2	3.1 ± 0.6
Fat mass (kg)	18.2 ± 7.4	19.5 ± 7.8	17.5 ± 7.3
Fat free mass (kg)	38.9 ± 5.6	39.0 ± 4.6	38.8 ± 6.2
Fat mass index (kg/m^2^)	7.0 ± 2.8	7.8 ± 3.1	6.6 ± 2.7
Fat free mass index (kg/m^2^)	15.1 ± 1.9	15.6 ± 1.6	14.9 ± 2.0

*Abbreviations:* SD – standard deviation TBW-total body water, L-litres.

### Physical activity

The mean VM/day was 859733 ± 319404 for rural and 740356 ± 222551 for urban women and the difference in physical activity among urban and rural women was not significant (*p* = 0.31). The mean wear time was 802 ± 118 minutes. There results indicate that there was no significant difference in the VM/day and wear time means of the rural and urban groups. The intensity of activity using Troiano et *al*. [22] cut-off points indicated that 44.5% of the activity was sedentary, 48.7% of the activity was light, 8% of the activity was moderate and 0.02% was vigorous (Table 3).

**Table 3:** **Physical activity among rural and urban women in Narok County, Kenya**

PA	Population mean SD*n*(22)	Rural*n*(8)	Urban*n*(14)
VM counts/day	783766 ± 261001	859733 ± 319404	740356 ± 222551
Sedentary^∞^	359 ± 120	352 ± 89.5	364 ± 137.3
Light^∞^	388 ± 87	377 ± 78.6	394 ± 92.9
Moderate^∞^	71 ± 45	80 ± 50.2	65.6 ± 42.6
Vigorous^∞^	0.18 ± 0.34	0.32 ± 0.48	0.10 ± 0.20
Total wear time^∞^	802 ± 118	800 ± 93.1	803.4 ± 133.9
**Activity levels**	**Population (%)**	**Rural (%)**	**Urban (%)**
Sedentary^∞^	44.5	42.5	44.3
Light^∞^	48.7	46.9	47.7
Moderate^∞^	8.8	10.5	7.9
Vigorous^∞^	0.02	.03	.02

VM- vector magnitude, ^∞^ time in minutes based on cut-off points by Troiano et *al*. [22].

### Regression results

Linear regression results showed a positive significant relationship between water turnover and BM (r^2^ = .38, *p* < 0.001) and BMI (r^2^ = .45, *p* < 0.001, *n* = 28). With regard to body composition, water turnover was positively associated with FMI (r^2^ = .41, *p* < 0.001, n = 28) and FFMI (r^2^ = .18, *p* < 0.05, n = 28). Water turnover was also positively associated with physical activity (r^2^ = .25 *p* < 0.05, n = 22) but not associated with wear time.

Multiple linear regression analysis revealed that including both physical activity and BMI in the model resulted in a 15% increase in explained variation in water turnover (r^2^ = .53, *p* < 0.05; ∆r^2^ = .15, *p* < 0.05, *n* = 22) compared to BMI alone (r^2^ = 0.38, *p* < 0.005 n = 22). The standardized beta values for BMI and physical activity were 0.544 and 0.393 respectively indicating that BMI was the strongest predictor of water loss in this model (Table 4). FMI alone explained 37% of the variation in water loss (r^2^ = .37, *p* < 0.005, *n* = 22) and adding physical activity to the model explained an additional 12% (r^2^ = .49, *p* = 0.05; ∆r^2^ = .12, *p* < 0.05). The standardized beta values were .509 for FMI and .359 for physical activity, indicating that FMI was the stronger predictor in this model (Table 5). In Table 6, both physical activity together with FFMI in the model resulted in an additional variation of 26% in water loss (r^2^ = .40, *p* > 0.01, ∆r^2^ = .26 *p* < 0.05) compared to FFMI alone (r^2^ = .14, *p* = 0.08, *n* = 22). Each one unit increase of BMI brings about 0.11 L/d increase in water loss holding physical activity constant, whereas a unit increase in FFMI brings about 0.17 L/d increase in water loss holding physical activity constant. Wear time in addition to physical activity did not significantly contribute to the variation in water loss.

**Table 4:** **Multiple regression results of water turnover (L/day) as dependent variable and BMI and VM/day as independent variables (n = 22)**

L/day	B	SE	β	*P*
**Step 1**				
Constant	.437	.797		
BMI	.122	.037	.62	<0.005
**Step 2**				
Constant	-.181	.755		
BMI	.107	.034	.54	<0.005
VM/day	1.219E-006	.000	.39	<0.05

Note Step 1: R^2^ = .38 (*p* < 0.005); Step 2: R^2^ = .53 (p < 0.05), R^2^ ∆ from step 1 = 0.15: L/day = liters per day, BMI = Body mass index, VM/day = Vector magnitude counts per day.

**Table 5:** **Multiple regression of water turnover (L/day) as dependent variable and FMI and VM/day as independent variables (n = 22)**

L/day	B	SE	β	*P*
**Step 1**				
Constant	1.988	.383		
FMI	.163	.048	.61	<0.005
**Step 2**				
Constant	1.311	.478		
FMI	.137	.046	.51	<0.01
VM/day	1.113E-006	.000	.36	<0.05

Note Step 1: R^2^ = .37 (*p* < 0.005), Step 2: R^2^ = .49 (*p* < 0.05), R^2^∆ from Step 1 = .12: L/day = liters per day, FMI = Fat mass index, VM/day = Vector magnitude counts per day.

**Table 6:** **Multiple regression of water turnover (L/day) as dependent variable and FFMI and VM/day as independent variables (n = 22)**

L/day	B	SE	β	*P*
**Step 1**				
Constant	.650	1.408		
FFMI	.167	.092	.38	NS
**Step 2**				
Constant	-.699	1.294		
FFMI	.174	.078	.39	<0.05
VM/day	1.580E-006	.000	.51	<0.01

Note Step 1: R^2^ = .14 (*p* = NS), Step 2: R^2^ = .40 (*p* < 0.01), R^2^∆ from Step 1 = .26: L/day = liters per day, FFMI = Fat free mass index, VM/day = Vector magnitude counts per day, NS = not significant.

## Discussion

Our study objective was to assess water loss and its relationship with physical activity and body composition among women in rural and urban dwellings in Narok County, Kenya. We found a positive relationship between body composition (BMI, FFMI, and TBW) and water turnover. Physical activity was also a positive predictor of water loss and physical activity together with BMI explained a 53% variation in water loss. Water loss in our study was comparable to the findings in other studies done in varied climatological conditions [6–8].

Water is an important nutrient in the maintenance of life and a major constituent of the human body. Water loss in itself is obligatory for elimination of wastes such as urea and excess salts via the kidney and carbon dioxide from the lungs and thermoregulation by way of sweat [23]. Water needs are a challenge to meet in sub-Saharan Africa, where food and water can be scarce and some communities in arid and semi-arid lands have to travel for several miles in search of both [24]. Numerous factors, such as high temperature, humidity levels, physical activity and exercise, and heat stress in particular influence water needs [23]. The average temperatures in our study area during the March – May season were between 14°C and 24°C for minimum and maximum respectively, whereas the average relative humidity levels are between 40% and 98% for the lowest to highest respectively [25]. March to May is the wet rainy season in Narok county and the temperatures during the dry season range from 20°C to 34°C [26]. High humidity in a hot climate makes it hard for the body’s natural cooling mechanism of sweating to take place and the body compensates by working harder to cool off and overheating or heat exhaustion can occur [27]. This may lead to dehydration and chemical imbalance within the body [27].

Thus, for women in most Kenyan communities who have the responsibilities of being key providers for their families and especially children, if water needs are not met, their nutritional status may be compromised since water is an essential nutrient [28] and their ability to provide for the household will be limited due to dehydration which causes impaired physiological and performance responses [28]. In a Dutch study, it was found that water turnover among women was similar to our study and was not related to physical activity [6]. The magnitude of water loss through sweat during exercise in a warm environment is reported to be dependent primarily on exercise intensity and duration [29]. Adequate total water intakes for sedentary adults under temperate climatic condition on average are between 2 and 2.5 L/day for women and men respectively and water loss for a sedentary adult is between 2–3 L/day [23], whereas for those in hot environments this need increases variably depending on prevailing conditions, some studies indicate up to 6 L/day [5, 30]. However, studies have shown that human water requirements should not be based on one meeting the minimal intake for age and gender because this can lead to deficit and possible adverse performance and health consequences [5]. The women in our study group participated mainly in sedentary and light activities, however, due to the mostly high temperatures the activity levels may be intense for a short period of time and during the rest of the day the activity intensity levels may slow down and remain low throughout. However, physical activity in most sub-Saharan African communities like most developing countries comes as part of the daily activities of searching for water and food [31, 32]. Rarely do adults exercise particularly in the rural areas unless for professional athletes and sports personalities. In comparison to a study by our group done on young adults (18-28 yrs) in the Netherlands (*n* = 40; unpublished data) - on average the Dutch subjects spent 66.9% of their waking time per day engaged in sedentary behavior compared to the Kenyans who spent 44.5%. The Kenyan subjects spent twice as much of their time on moderate intensity PA (8.8%) compared to the Dutch (4.4%). The average vector magnitude counts per day for the Kenyan subjects were 783766 while the average for the Dutch subjects was 524206 vector magnitudes per day. Using the cut-off points by Freedson for adults, our subjects falls within the light category of physical activity [33].

Overweight existed among women in the study area and although our sample size was small to give us an estimated prevalence, the trends of increasing overweight cases in Africa is worrying and previous studies have shown that overweight impedes physical activity and movement [34]. In our study, we found the percentage of FM to be higher and FFM to be lower compared to that reported in a study done in another part of Kenya [17]. The study found the percentage body fat among lactating women to be 26.1% compared to our study which was 30.7% and FFM was 41.3 kg while in our study it was 38.9 kg. Another study among women in Kenya also found the mean TBW to be 24.0 L [35], while in our study the mean was 29.3.L. The TBW in our study was comparable to that found in studies in the US [36] and Kenya [17]. FMI and FFMI correlated significantly with water turnover. LBM is about 73% water and fat body mass is 10% water [5, 37] and TBW and LBM have been reported to strongly correlate with body surface area in normal and obese subjects [38], therefore presumably individuals with higher BMI would have higher water requirements and therefore water turnover is expected to be higher as BMI increases, as it was in our study and these findings concur with a study among overweight adolescents [14].

Despite varying water needs brought about by differences in climatic conditions, physical activity levels, metabolic rate, body surface area and body weight and body composition [39], healthy humans regulate their water balance with precision [23]. Meeting water requirements among women is essential for proper normal body function [40] in order for them to accomplish cultural responsibilities of caring for household members. Heat acclimation may play a role in water regulation [41, 42] and individuals living in hot climates experience physiological thermoregulation in order for the body to utilize water well [42]. Water loss among our study population was more or less similar to those of women in other climatic regions. Studies have indicated that well acclimatized humans in extremely warm/hot climates can sustain a perspiration rate of 2 L/hour for long duration activity [43] and another study indicates that for short time periods a sweating rate of 4 L/hour has been reported [44]. However humans have limited rehydration capacity which allows about 1 L at a time such that even if water is readily available, those working in warm climates will drink less than that lost through perspiration concluding that activity levels were not directly tied to and limited by water availability [45]. Our recommendation for future research is the need for more research on physical activity, water requirement and regulation as well as coping mechanism among women in sub-Saharan Africa with a larger study population to offer representative data. Our study was limited by the small sample size and care must be exercised while making generalizations. However, the strength of our study is in the methodology; we used deuterium dilution to assess body composition and water turnover, whereas accelerometers were used to assess physical activity; methods that have been validated in previous studies and have been found to be reliable.

## Conclusion

In conclusion, water turnover among women was related to physical activity and body size. BMI together with physical activity were the strongest predictors of water loss among our study group. There was no significant difference in physical activity, body composition and water turnover among rural and urban women.
